# Supplementary material for: Analysis of PPARγ Signaling Activity in Psoriasis
Source: Int J Mol Sci. 2021 Aug 10;22(16):8603. doi: 10.3390/ijms22168603 (PMC8395241; doi:10.3390/ijms22168603)
Supplement: Supplementary file 1 [file ijms-22-08603-s001.zip › Supplemental materials_Analysis of PPARg signaling activity in psoriasis/Pathway models/Models images and html files/PPARG inhibits phenotypes assotiated with psoriasis/191721.html]

T-helper 17 cell differentiation


# Cell Process T-helper 17 cell differentiation

|  |  |
| --- | --- |
| URN | urn:agi-gocellproc:0072539 |
| Total Entities | 0 |
| Connectivity | 553 |
| Name | T-helper 17 cell differentiation |

---

|  |  |
| --- | --- |
| Pathway | AHR Signaling in Th17 Cells Function |
|  | Natural Killer T-Cell Roles in Diabetes Mellitus Type 1 |
|  | IDO1 in Cancer Immune Escape |
|  | PPARG inhibits phenotypes assotiated with psoriasis |
|  | efects selected |

---

|  |  |
| --- | --- |
| MedScan ID | 4159118 |

---

|  |  |
| --- | --- |
| Alias | T-helper 17 cell differentiation |
|  | T-helper 17 cell development |
|  | development of T helper 17 cells |
|  | differentiation of T helper 17 cells |

---

|  |  |
| --- | --- |
| GO ID | 0072539 |

---
